# Divergent and Overlapping Roles for Selected Phytochemicals in the Regulation of Pathological Cardiac Hypertrophy

**DOI:** 10.3390/molecules26051210

**Published:** 2021-02-24

**Authors:** Levi Evans, Yiqui Shen, Abigail Bender, Leah E. Burnett, Musheng Li, Justine S. Habibian, Tong Zhou, Bradley S. Ferguson

**Affiliations:** 1 Department of Nutrition, University of Nevada, Reno, NV 89557, USA; levi659@gmail.com (L.E.); nvlvchristine@gmail.com (Y.S.); abigail_bender@yahoo.com (A.B.); lburnett@nevada.unr.edu (L.E.B.); 2 Environmental Sciences Program, University of Nevada, Reno, NV 89557, USA; 3 Department of Pharmacology and Physiology, University of Nevada, Reno, NV 89557, USA; mushengl@unr.edu (M.L.); tongz@med.unr.edu (T.Z.); 4 Cellular and Molecular Biology, University of Nevada, Reno, NV 89557, USA; jhabibian@nevada.unr.edu; 5 Center of Biomedical Research Excellence for Molecular and Cellular Signal Transduction in the Cardiovascular System, University of Nevada, Reno, NV 89557, USA

**Keywords:** phytochemical, emodin, berberine hydrochloride, apigenin, baicalein, pathological cardiac hypertrophy, nutrient-gene expression

## Abstract

Plant-based foods, like fruits, vegetables, whole grains, legumes, nuts, seeds and other foodstuffs, have been deemed as heart healthy. The chemicals within these plant-based foods, i.e., phytochemicals, are credited with protecting the heart. However, the mechanistic actions of phytochemicals, which prevent clinical endpoints, such as pathological cardiac hypertrophy, are still being elucidated. We sought to characterize the overlapping and divergent mechanisms by which 18 selected phytochemicals prevent phenylephrine- and phorbol 12-myristate 13-acetate-mediated cardiomyocyte enlargement. Of the tested 18 compounds, six attenuated PE- and PMA-mediated enlargement of neonatal rat ventricular myocytes. Cell viability assays showed that apigenin, baicalein, berberine hydrochloride, emodin, luteolin and quercetin dihydrate did not reduce cell size through cytotoxicity. Four of the six phytochemicals, apigenin, baicalein, berberine hydrochloride and emodin, robustly inhibited stress-induced hypertrophy and were analyzed further against intracellular signaling and genome-wide changes in mRNA expression. The four phytochemicals differentially regulated mitogen-activated protein kinases and protein kinase D. RNA-sequencing further showed divergence in gene regulation, while pathway analysis demonstrated overlap in the regulation of inflammatory pathways. Combined, this study provided a comprehensive analysis of cardioprotective phytochemicals. These data highlight two defining observations: (1) that these compounds predominantly target divergent gene pathways within cardiac myocytes and (2) that regulation of overlapping signaling and gene pathways may be of particular importance for the anti-hypertrophic actions of these phytochemicals. Despite these new findings, future works investigating rodent models of heart failure are still needed to understand the roles for these compounds in the heart.

## 1. Introduction

Plants, both edible and not, have been shown to protect the heart [1]. In fact, several meta-analyses have reported that diets rich in fruits, vegetables, whole grains, nuts, seeds and other plant-based foods found in vegan/vegetarian diets, the Mediterranean Diet or the DASH diet (Dietary Approaches to Stop Hypertension) reduce the risk of heart diseases including heart failure [2,3,4]. Certainly, a heart-healthy diet is one consisting of a wide variety of plants [4]. Additionally, plant-based remedies have been used in Traditional Medicine practices for centuries; food bioactives, or phytochemicals, contained in these traditional medicines and plants have been shown to attenuate pathological cardiac hypertrophy [5] and are thus often credited for cardioprotection.

Phytochemicals are synthesized by plants to aid in protection against UV rays, pathogens and predators, but they can also interact in human biological processes after consumption. Historically, biomedical research focused on the actions of phytochemicals in reduction-oxidation reactions and immunological signaling [6]. In these early reports, phytochemicals offered cardioprotection through attenuated pathological cardiac hypertrophy by reducing oxidative damage and attenuating inflammation [6]. More recent evidence, however, demonstrates that phytochemicals can regulate other mechanisms to inhibit pathological cardiac hypertrophy that includes regulation of signal transduction (e.g., mitogen activated protein kinases (MAPK)), contractile function (e.g., Ca^2+^ handling) and epigenetic modifications (e.g., histone deacetylase (HDAC) inhibition) [7]. As such, phytochemicals may act as natural pharmacological agents that prevent pathological enlargement of the heart through a myriad of cellular changes.

In this report, we examined the inhibitory actions of eighteen phytochemicals in a cellular based model of pathological cardiac hypertrophy. Of these 18 screened phytochemicals, six—apigenin; baicalein; berberine hydrochloride (BHCL); emodin (Emod); luteolin (Lut) and quercetin dihydrate (QD)—inhibited pathological cardiac hypertrophy in response to two distinct agonists (phenylephrine (PE) and phorbol 12-myristate 13-acetate (PMA)). Potent inhibition (i.e., >80%) was noted for four of these phytochemicals: apigenin; baicalein; BHCL and emodin. As mentioned above, phytochemicals are pleiotropic. Consistent with this, we report that these four phytochemicals which potently inhibited pathological cardiac hypertrophy regulated overlapping as well as divergent changes in global gene expression and signal transduction pathways.

## 2. Results

### 2.1. Six Phytochemicals Attenuated Pathological Cardiac Hypertrophy

Pathological cardiac hypertrophy is characterized by enlarged and weakened cardiomyocytes and has been shown to impair heart function and to result in heart failure. Likewise, inhibition of pathological cardiomyocyte hypertrophy has been shown to improve heart function [8,9]. Here, we sought to identify phytochemicals that could inhibit pathological cardiac hypertrophy. As such, we screened 18 selected phytochemicals in a model of pathological cardiac hypertrophy: apigenin; baicalein; baicalin; BHCl; caffeic acid; dihydromyricetin; emodin; epigallocatechin gallate; gossypol; hematoxylin; indirubin; kaempferol; luteolin; morin hydrate; myricetin; myricitrin; palmatine; and QD. These 18 phytochemicals and their doses were selected as we previously observed that all of these compounds could inhibit histone deacetylase (HDAC) activity in vitro [10]; HDAC inhibition attenuates pathological cardiac hypertrophy [11]. For these studies, we induced hypertrophy in neonatal rat ventricular myocytes (NRVMs) by stimulating with either an α1-adrenergic receptor agonist (phenylephrine; PE) or an intracellular agonist (phorbol 12-myristate 13-acetate; PMA) prior to co-spiking with one of 18 dietary compounds. NRVMs were also treated with our vehicle (Veh; DMSO) control. Baicalin, caffeic acid and dihydromyricetin did not attenuate cardiomyocyte hypertrophy under any condition (Table 1). Gossypol and myricetin appeared toxic as NRVMs treated with these two compounds died in culture. Hematoxylin, kaempferol, myricitrin and morin hydrate blocked PMA-induced hypertrophy with no effect noted for PE-induced hypertrophy; hematoxylin was the only compound with considerable inhibitory actions (Table 1). In contrast, indirubin and palmatine only blocked PE-induced cardiomyocyte hypertrophy (Table 1). Epigallocatechin (EGCG) showed little effect on either hypertrophic agonist (Table 1).

**Figure 1:**
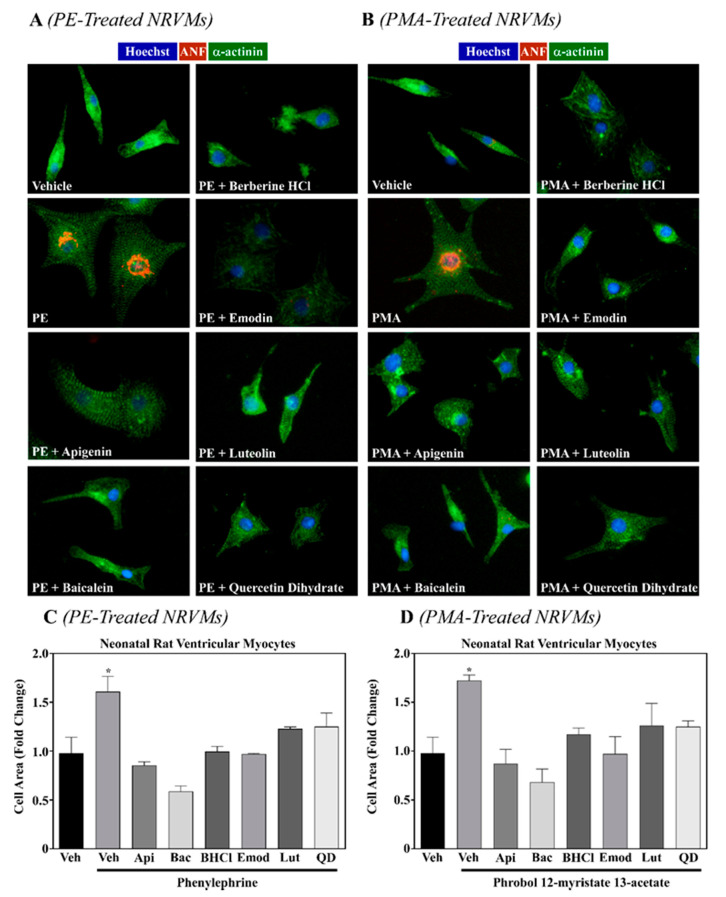
Select phytochemicals attenuate cardiac hypertrophy in response to PE or PMA. NRVMs were stimulated to hypertrophy with (**A**) phenylephrine (PE; 10 µM) or (**B**) phorbol 12-myristate 13-acetate (PMA; 50 nM) in the absence or presence of vehicle control or phytochemicals (Apigenin (Api), Baicalein (Bac), berberine hydrochloride (BHCL), Emodin (Emod), Luteolin (Lut), or Quercetin dehydrate (QD); All compounds were used at 10 µM). NRVMs were co-treated as indicated above and cells were fixed 48 h post-treatment and stained for α-actinin, atrial natriuretic peptide (ANP), and Hoescht. NRVMs were visualized with the EVOS FL microscope and (**C**) PE and (**D**) PMA treated cells quantified using Image J software and graphed with GraphPad Prism software. One-way ANOVA with Tukey’s post-hoc was performed and significance set at *p* < 0.05. *—Significantly different than all treatment groups.

**Figure 2:**
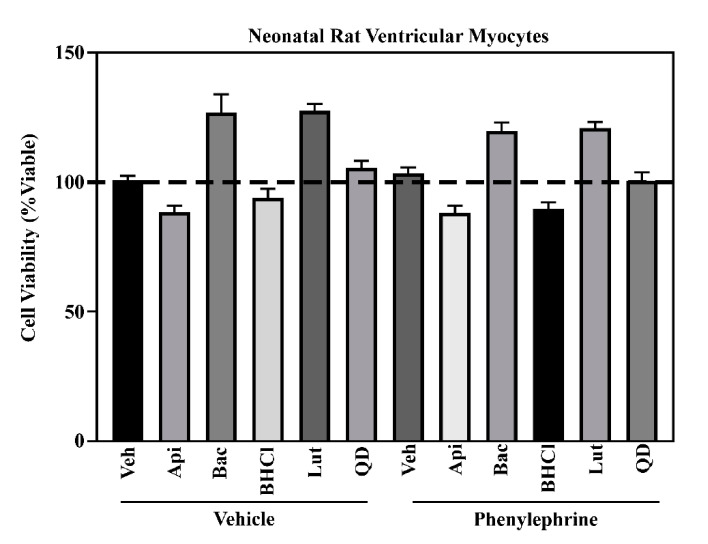
Phytochemical-treated NRVMs show no signs of cytotoxicity. Neonatal rat ventricular myocytes (NRVMs) were treated with the indicated phytochemicals (Apigenin (Api), Baicalein (Bac), berberine hydrochloride (BHCL), Luteolin (Lut), or quercetin dehydrate (QD); All compounds were used at 10 µM) for 48 h in the absence or presence of phenylephrine (10 µM) prior to alamarBlue™ assay. A One-way ANOVA with Tukey’s post-ho was used to asses significance (*p* < 0.05).

Of the 18 compounds screened, apigenin, baicalein, BHCl, emodin, luteolin and QD were the only compounds that attenuated pathological cardiac hypertrophy in NRVMs exposed to PE or PMA (Table 1; Figure 1). It is important to note that inhibition of hypertrophy was not due to cytotoxicity, as these six phytochemicals did not decrease cell viability (Figure 2). While luteolin and QD did inhibit pathological hypertrophy (>40% inhibition), apigenin (>90%), baicalein (>90%), BHCl (>50%) and emodin (>75%) demonstrated the strongest anti-hypertrophic actions (Table 1; Figure 1). As such, we sought to further characterize the overlapping and divergent mechanistic actions of these four phytochemicals in the cardiomyocyte.

### 2.2. Phytochemicals Regulate Overlapping and Divergent Changes in Gene Expression

**Figure 3:**
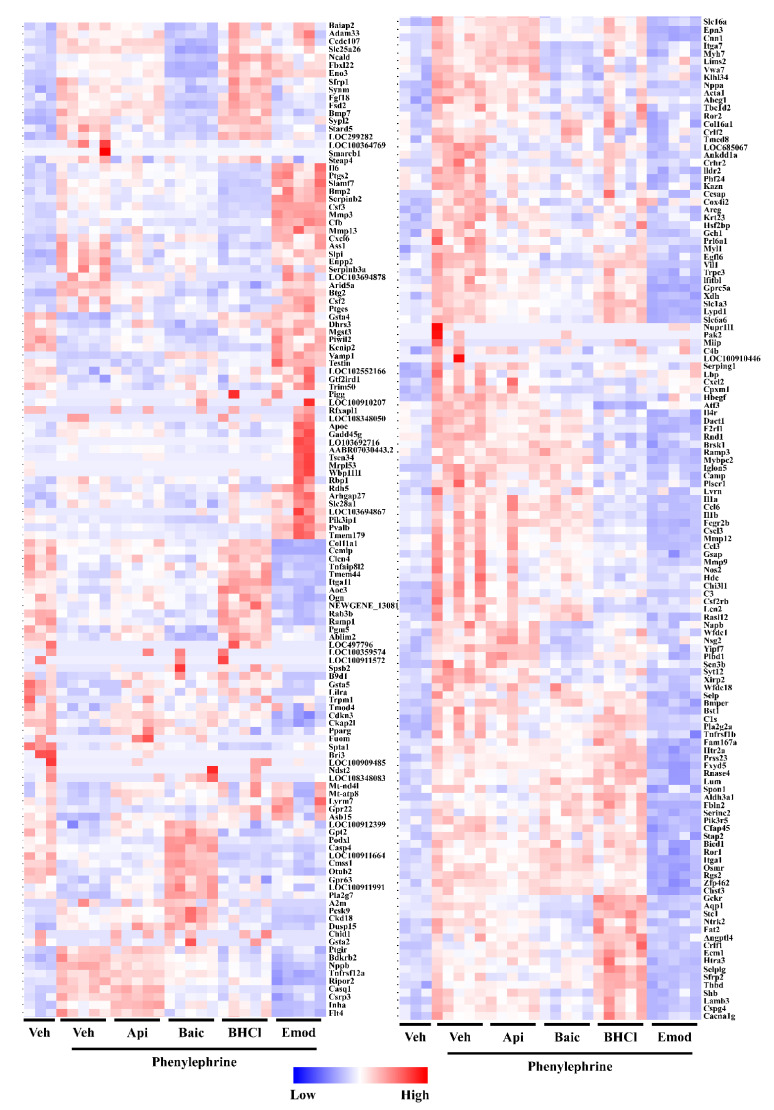
Phytochemicals regulate divergent and overlapping gene pathways in response to PE-induced cardiac hypertrophy. NRVMs were stimulated to hypertrophy with phenylephrine (PE; 10 µM) in the absence or presence of vehicle control or phytochemicals (Apigenin (Api), Baicalein (Bac), berberine hydrochloride (BHCL), Emodin (Emod), Luteolin (Lut), or quercetin dehydrate (QD); All compounds were used at 10 µM) for 48 h. RNA was isolated and sequenced with the NEXT-Seq 500. Differential gene expression was visualized via heatmap and the expression level was scaled for each gene. Additional information for each gene can be found in Supplemental Table S1.

**Figure 4:**
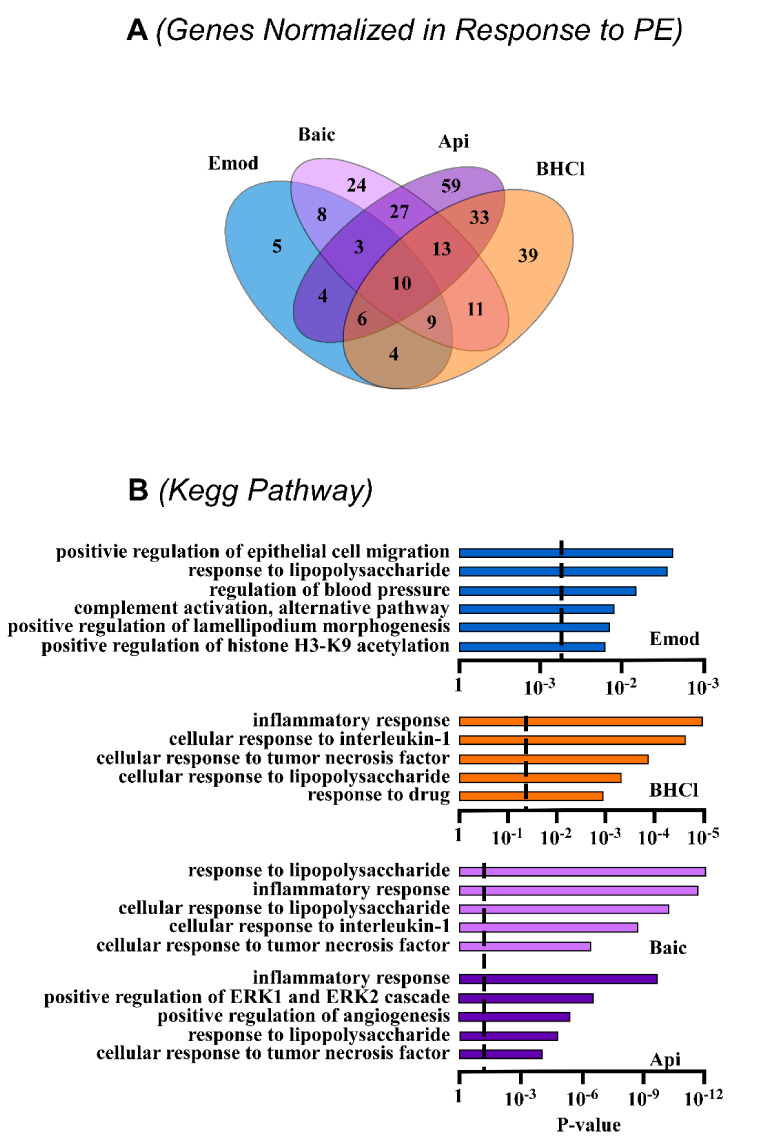
Phytochemicals regulate divergent and overlapping gene pathways in response to PE-induced cardiac hypertrophy. NRVMs were stimulated to hypertrophy with phenylephrine (PE; 10 µM) in the absence or presence of vehicle control (Veh) or phytochemicals (apigenin (Api), baicalein (Bac), berberine hydrochloride (BHCL), or emodin (Emod); All compounds were used at 10 µM) for 48 h. RNA was isolated and sequenced with the NEXT-Seq 500. (**A**) A Venn diagram was used to show genes normalized by phytochemical treatment. (**B**) KEGG pathway analysis was used to examine the top five pathways regulated by the select phytochemicals in response to PE.

**Figure 5:**
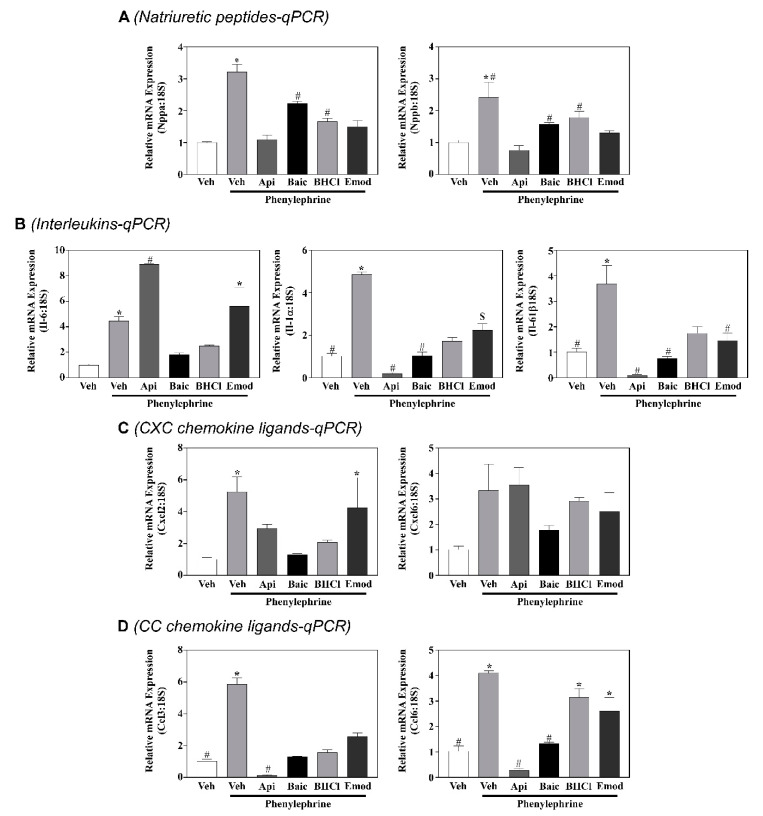
Phytochemicals regulate hypertrophic and inflammatory gene expression in NRVMs. Neonatal rat ventricular myocytes (NRVMs) were stimulated to hypertrophy with phenylephrine (PE; 10 µM) in the absence or presence of vehicle control (Veh) or phytochemicals (apigenin (Api), baicalein (Bac), berberine hydrochloride (BHCl), emodin (Emod); All compounds were used at 10 µM) for 48 h. RNA was isolated and qPCR performed to analyze the (**A**) natriuretic peptides (ANP and BNP), (**B**) interleukins (IL-6, IL-1α, and IL-1β, (**C**) CXC chemokine ligands (CXCL2 and CXCL6) and (**D**) CC Chemokine ligands (CCL3 and CCL6). 18S rRNA was used as our endogenous control and gene expression expressed using 2^−ΔΔCt^. One-way ANOVA with Tukey’s post-hoc was used to determine significance (*p* < 0.05). *—Significantly different from values not marked with an asterick, #—significantly different from phenylephrine, S—significantly different from apigenin.

Data above showed that apigenin, baicalein, BCHL and emodin attenuated pathological hypertrophy more robustly than luteolin and QD. As a next step, we sought to identify gene pathways regulated by our compounds in myocytes in response to PE. For this, RNA-sequencing was used to help elucidate potential mechanistic targets for phytonutrient actions. As expected, PE significantly altered gene expression, and these actions were normalized by apigenin, baicalein, BHCl and emodin (Figure 3; Supplemental Table S1). However, it should be noted that while there was overlap for some of these normalized genes, our compounds divergently regulated gene expression profiles as observed by our heatmap (Figure 3) and Venn diagram (Figure 4A). Specifically, all four compounds similarly normalized 10 genes in hypertrophied myocytes (Figure 4A). In contrast, apigenin independently normalized 59 genes, baicalein 24 genes, BHCl 39 genes and emodin 5 genes (Figure 4A); these data clearly demonstrate divergence in gene regulation by phytochemicals despite similar phenotypic outcomes. Despite this divergence, pathway analysis highlighted that apigenin, baicalein, BHCl and emodin normalized genes involved in the inflammatory process (Figure 4B). In fact, the top primary pathway affected by apigenin and BHCl was the inflammatory response and for baicalein lipopolysaccharide (Figure 4B). Nevertheless, individual proinflammatory genes, including interleukin (IL)-1α, -1β and -6, chemokine (C-X-C motif) ligand (CXCL) 2 and 6 as well as chemokine (C-C motif) ligand (CCL) 3 and 6, were differentially regulated by the four phytochemicals (Figure 5). Consistent with our RNA-seq data sets, qPCR validation showed that PE induced the expression of ANP and BNP as well as the interleukins and chemokines, except for CXCL6 which was not significantly increased (Figure 5). In addition, we report that all of the phytochemicals inhibited ANP and BNP expression, with apigenin eliciting the greatest response (Figure 5); these findings were consistent with our RNA-seq data. Similarly, we our RNA-seq (Figure 3 and Supplemental Table S1) and qPCR (Figure 5) data show that PE-induced IL-1α and IL-1β are attenuated by all four phytochemicals, with apigenin inhibiting IL-1 expression below baseline, while emodin attenuates IL-1 expression back to baseline. Interestingly, our RNA-seq (Figure 3 and Supplemental Table S1) and qPCR (Figure 5) data further showed that PE-induced IL-6 was attenuated by baicalein, BHCL, and emodin, with but induced by apigenin (Figure 5). While CXCL12 qPCR data supports our RNA-seq findings, CXCL6 is inconsistent as RNA-seq data (Figure 3 and Supplemental Table S1) suggest that this chemokine is significantly induced by PE and attenuated by our phytochemicals; here our qPCR data suggests that while there is a nonsignificant increase in CXCL6 only baicalein appears to attenuate expression although not significantly (Figure 5). It should be noted however that qPCR examination of the CCL chemokines CCL3 and CCL6 (Figure 5) match our RNA-seq (Figure 3 and Supplemental Table S1) data sets in which PE-induced CCL3 was attenuated by all of our phytochemicals while PE-induced CCL6 was only attenuated by apigenin and baicalein. Together, these data strengthen the postulate that phytochemicals can regulate divergent genes, and can also work through overlapping pathways to attenuate cardiac myocyte hypertrophy. Still, not all pathways overlapped. For example, apigenin regulated ERK1 and ERK2 cascades, while emodin was predicted to regulate histone H3-K9 acetylation based on gene changes (Figure 4B); these pathways were not observed with the other phytonutrients.

### 2.3. Phytochemicals Regulate Overlapping and Divergent Changes in Signal Transduction

**Figure 6:**
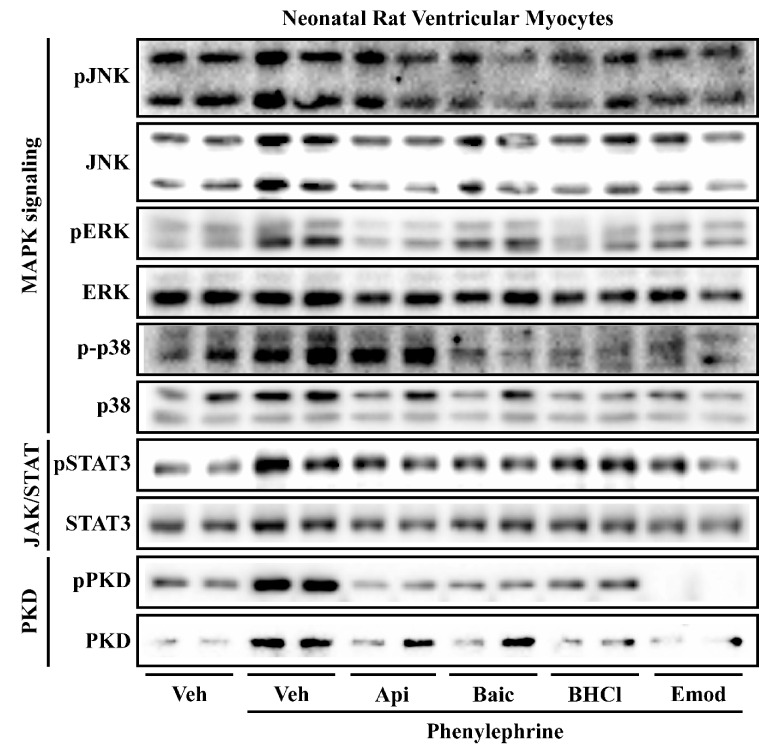
Phytochemicals regulate overlapping and divergent signaling pathways in NRVMs in response PE-induced hypertrophy. Neonatal rat ventricular myocytes (NRVMs) were treated with vehicle (Veh) or phenylephrine (PE; 10 µM) in the absence or presence of phytochemicals (apigenin (Api); baicalein (Bac); berberine hydrochloride (BHCL), or emodin (Emod)). NRVMs were co-treated with PE or 10 µM of specified phytochemicals for 48 h prior to cell lysis and immunoblot analysis for MAPK signaling (phospho-ERK, total ERK, phospho-JNK, total JNK, phospho-p38, total p38), JAK/STAT signaling (phospho-STAT3, total STAT3) and PKD signaling (phospho-PKD, and total PKD). Immunoblots were imaged with the BioRad Chemi-Doc XRS+.

**Figure 7:**
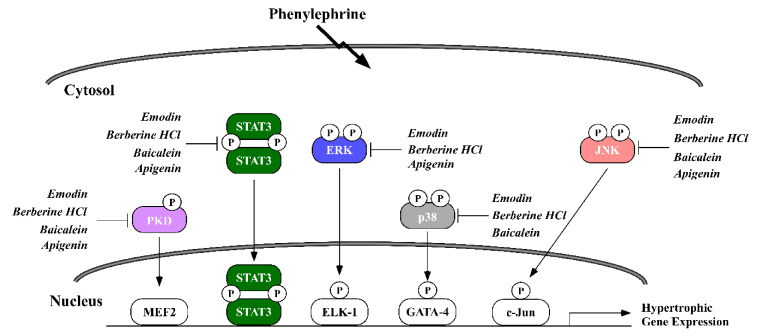
Model showing which signaling pathways were targeted by phytochemical treatment.

Several signal transduction pathways participate in causing the cardiomyocyte to hypertrophy. Phosphorylation of the mitogen activated protein kinases (MAPKs), Janus kinase-signal transducer and activator of transcription (JAK/STAT) and protein kinase D (PKD) pathways are linked to pathological heart enlargement and inflammation [12,13,14]. Thus, we examined how apigenin, baicalein, BHCl and emodin would regulate MAPKs, PKD and JAK/STAT in NRVMs exposed to PE. Our data showed that PE increased MAPK phosphorylation for c-Jun N terminal kinase (JNK), extracellular regulated-signaling kinase (ERK) and p38 MAPK (p38) as well as PKD phosphorylation and STAT3 phosphorylation (Figure 6). Apigenin reduced JNK and ERK phosphorylation but not p38 (Figure 6). Baicalein reduced JNK and p38 phosphorylation but had little effect on ERK phosphorylation (Figure 6). Both BHCl and emodin reduced phosphorylation of all MAPKs (Figure 6). It is important to note that the phytochemicals did not attenuate total ERK, JNK or p38 protein expression. All the phytochemicals reduced PKD phosphorylation and total PKD; however, emodin completely abolished PKD phosphorylation (Figure 6). Apigenin, baicalein, BHCl and emodin minimally reduced total STAT3 expression as well as STAT3 phosphorylation. It should be noted that Coomassie staining demonstrated that total protein expression remained unchanged with treatment (Supplemental Figure S1). Consistent with our gene expression, these data suggest that apigenin, baicalein, BHCl and emodin can prevent agonist-induced cardiomyocyte hypertrophy through divergent and overlapping changes in signal transduction (Figure 7).

## 3. Discussion

In this study, we screened 18 phytochemicals to identify compounds that would inhibit pathological cardiomyocyte hypertrophy (Table 1). We report that six phytochemicals: apigenin; baicalein; BHCl; emodin; luteolin and QD inhibited cardiomyocyte hypertrophy in response to two distinct agonists, PE or PMA (Table 1, Figure 1). Further examination of these compounds demonstrated overlapping and divergent regulation of genome-wide changes in gene expression and signal transduction. These findings are consistent with the fact that phytochemicals elicit a myriad of changes within cells that involve changes in oxidation-reduction, inflammation, gene expression and intracellular signaling [15,16,17]. Thus, our compounds likely regulate similar and divergent pathways to improve cardiac myocyte size; although it remains unclear if these compounds will work to improve cardiac remodeling and function in vivo. Still, these data suggest that phytochemicals found in fruits and vegetables elicit a multi-faceted response for myocardial disease protection. In addition, our findings show that while these four phytochemicals predominantly target divergent gene pathways, there is considerable overlap in signal transduction; this overlapping regulation potentially drives the anti-hypertrophic phenotype common for these compounds. 

Our experiments elicited some notable findings, including stronger anti-hypertrophic actions between phytochemical derivatives (e.g., the aglycone baicalein versus its glycoside baicalin) and phytochemical families (e.g., the flavones apigenin and luteolin versus the flavonols quercetin and kaempferol). Indeed, baicalein is the aglycone of baicalin, both of which are found in the *Scutellaria baicalensis* Georgi plant, and our data show that baicalein inhibited agonist-induced cardiomyocyte hypertrophy more so than baicalin. This is interesting as some studies report that baicalin prevents pathological cardiac hypertrophy, fibrosis and dysfunction in transverse aortic constricted mice [18], and others report that baicalin in combination with other phytochemicals is anti-hypertrophic [19]. These data contrast our findings which suggest that baicalein inhibits hypertrophy whereas baicalin does not block pathological hypertrophy in primary cardiomyocytes. In agreement with our data, baicalein has been shown by others to attenuate cardiac hypertrophy [20,21,22,23]. This could be for a variety of reasons that includes metabolism in vivo of baicalin or, as mentioned by Wu et al., baicalin-induced changes in the microbiome that would not take place in cell culture [19]. The microbiome participates in second phase metabolism of phytochemicals, which would subsequently synthesize systemically bioactive compounds and metabolites that could inhibit cardiac hypertrophy and not the parent compound. With the emergence of the microbiome in nutrition research, future research will likely yield new findings for phytochemical metabolism in cardioprotection. Baicalein may also have shown better cardioprotective properties than baicalin due to aglycones often having stronger bioactivity than their glycosides, though this is not always the case [24].

Regarding the anti-hypertrophic actions of flavones versus flavonols, our data show apigenin attenuated agonist-induced cardiomyocyte hypertrophy to a greater extent than quercetin and kaempferol. These data are surprising as flavonols like quercetin and kaempferol are suggested to be more bioactive than flavones like apigenin, likely due to the extra hydroxyl group in position three of the flavonol’s C-ring or the overall total of hydroxyl groups [25]. Indeed, researchers have shown that quercetin inhibited angiotensin-II-induced cardiomyocyte hypertrophy [26] and kaempferol inhibited phenylephrine-induced cardiomyocyte hypertrophy [27] in immortalized cell lines. These discrepancies from our results may be due to differences in agonists or cell culture experiments. On the other hand, similar to our findings, apigenin compared to select flavonols had a stronger effect on molecular signaling [28,29] and a similar effect on phenotypes [30,31] associated with disease. While our study supports the notion that at least the flavone, apigenin, was more efficacious than the flavonols, quercetin and kaempferol, for pathological cardiomyocyte hypertrophy, animal experiments that examine these compounds side-by-side may elicit different results.

The polyphenolic chemical structure of plant compounds is credited for, and often predict, their pharmaco-like bioactivities. Indeed, and as implied above, the number of hydroxyl groups within the pharmacophores of plant compounds have been associated with higher bioactivity, especially flavonoids with hydroxyl groups located at the five and seven carbon of the “A” ring and the four prime carbon of the “B” ring [32,33]. Hydroxyl groups provide strong hydrogen-bonding via electron trading, which has been shown to be an important driver of interactions between phytochemicals and their cellular targets [34]. In this regard: berberine contains no hydroxyl groups; apigenin, baicalein and emodin contain only three hydroxyl groups; while luteolin contains an additional hydroxyl group on the three-prime carbon (four total) and quercetin contains two additional hydroxyl groups on the three and three-prime carbons (five total). Thus, our data counterintuitively showed that apigenin (at higher concentrations), baicalein, berberine and emodin more potently inhibited pathological cardiomyocyte hypertrophy which suggests that the number of hydroxyl groups within a phytochemical pharmacophore does not always predict their efficacy towards clinical endpoints. It may be that additional hydroxyl groups on the “A” ring, the lack of hydroxyl groups on the “C” ring or specific locations and proximity of hydroxyl groups allow for better bioactivity against the failing cardiomyocyte.

Non-phenyl rings of phytochemicals further allow pharmacologic functionality; as is the case with the oxygenated heterocyclic “C” ring of flavonoids (e.g., apigenin, baicalein, luteolin and quercetin), the di-oxygenated “A” and nitrogenated heterocyclic “C-D” rings of benzylisoquinoline alkaloids (e.g., berberine) as well as the di-oxo ketone ring of anthraquinones (e.g., emodin). Berberine also contains methoxy functional groups, as well as hydrochloride in our experiments which has been shown to strengthen electron trading and bioactivities [35]. Outside of hydrogen-bonding, the chemistry of these phytochemical functional groups characterized above allow for molecular interactions via Van Der Walls, hydrophobic, ionic, metal complexation, π-π and cation-π binding. These interactions may subsequently stabilize, destabilize, allosterically cause a conformational change in or competitively inhibit the molecular target of interest. Therefore, while the types, location and proximity of the functional groups within the phytochemical pharmacophore are important for their bioactivity towards molecular targets of interest [36], the molecular structure of the target is also important in predicting phytochemical interactions and their outcomes [34,37]. Computational docking studies used to predict phytochemical-molecular interactions [34,38,39] suggest that phytochemicals-molecular targets include intracellular signaling cascades and the control of transcription factors that regulate gene expression, consistent with our findings.

Not surprisingly then we reported that our phytochemicals could inhibit various members of the mitogen-activated protein kinase family (MAPKs), in particular ERK, JNK and p38 (Figure 7). Activation of all three MAPKs has been implicated in pathological cardiac hypertrophy [12]. Interestingly, recent reports have shown that threonine 188 (T188) auto-phosphorylation of ERK drives PE-induced nuclear ERK compartmentalization necessary for pathological hypertrophy and fibrosis in vitro and in vivo [40]. While we did not look at this site, it is likely that several of our compounds would target T188 phosphorylation for inhibition. Our data would suggest that inhibition of ERK is one mechanism by which apigenin, BHCl and emodin can inhibit hypertrophy and indeed, KEGG pathway analysis showed that apigenin regulates the ERK cascade. Similar to ERK, p38 is pro-hypertrophic [41], where p38 inhibition attenuated pathological cardiomyocyte hypertrophy and p38 activation promotes cardiomyocyte hypertrophy [42]. This is interesting to note, as some of our compounds (i.e., baicalein, BHCl and emodin) attenuated p38 signaling while others did not change p38 phosphorylation (i.e., apigenin). Unsurprisingly, JNK is also activated in models of cardiomyocyte hypertrophy [43,44] and again we report that apigenin inhibited JNK phosphorylation, while baicalein showed little effect. Lastly, protein kinase D (PKD) has also been implicated in cardiac hypertrophy [45], in which PKD1 was shown to phosphorylate class II histone deacetylases, which led to HDAC translocation out of the nucleus and induction of pro-hypertrophic genes [14]. Apigenin, baicalein, BHCl and emodin all attenuated PKD phosphorylation at serine 744/748 (S744/748) (Figure 7), in which S744/748 PKD phosphorylation confers activity [46]. Future studies will need to be performed to determine if phytochemical-mediated inhibition of cardiomyocyte hypertrophy is a direct result from changes in MAPK/PKD signaling, or if changes in MAPK/PKD signaling are an indirect effect due to changes in cardiomyocyte hypertrophy.

While intracellular signaling has been linked to maladaptive cardiac hypertrophy, inflammation is considered a major player in most forms of cardiovascular disease [47]. Certainly, atherosclerosis, which is the formation of plaque within arteriole walls, is partially driven by inflammation [48]. More importantly, inflammation has been shown to drive cardiac hypertrophy [49]. In fact, the pro-inflammatory cytokine, interleukin-6 has been shown in several models to be critical for pathological cardiac remodeling [13,50,51]. Not surprisingly, pathway analysis demonstrated that all four phytochemicals in this report normalized genes involved in the inflammatory or LPS pathway in cardiomyocytes. We additionally showed that the four phytochemicals differentially regulated interleukin-6 gene expression. Of further interest, signal transducer and activator of transcription 3 (STAT3) may be important for interleukin 6-mediated cardiac hypertrophy [13], which we showed both to be downregulated by baicalein and BHCl. Finally, proinflammatory chemokine ligands, including those with the C-X-C motif (CXCL) and the C-C motif (CCL), have been implicated in cardiac dysfunction and remodeling [52,53,54]. Indeed, CXCL-1 was strongly associated with cardiac pathogenesis in angiotensin II-infused mice [53]. These experiments showed that treating angiotensin II-infused mice with a chemokine inhibitor prevented cardiac inflammation, hypertrophy, fibrosis and dysfunction [53]. We report here that apigenin, baicalein, BHCl and emodin differentially regulated the expression of pro-inflammatory chemokines (i.e., CXCL-2 and 6 as well as CCL-3 and 6). Thus, our data are consistent with the majority of science which shows that phytochemicals are cardioprotective by attenuating deleterious inflammation. 

Our data present new findings for the role of selected phytochemicals in gene regulation and hypertrophy, yet we should note the limitations of our study. A primary limitation of this study is that these experiments were conducted in cultured cardiomyocytes and not in an in vivo biological system which would then expose the issues of poor phytochemical bioavailability. Indeed, the six phytochemical parent compounds studied above—apigenin, baicalein, berberine, emodin, luteolin and quercetin—have all been shown to have low bioavailability and to reach plasma concentrations only in the low nanomolar range [55,56,57,58,59,60], far below the pharmacological, micromolar range that we used for cardiomyocytes. Furthermore, phytochemicals undergo first and second phase metabolism, involving liver- and gut microbiota-based xenobiotic enzymes (e.g., glucuronidase, sulfatase and glutathione transferase), which then results in structurally and functionally different metabolite compounds that, instead of being distributed to tissues of interest such as the heart, are commonly allocated to metabolizing and excretory tissues such as the liver, kidneys and lungs [58,61,62]. Studies which identify primary bioactive phytochemical metabolites and their distribution to target tissues are currently ongoing but, nevertheless, are in their infancy. Metabolomics coupled with other sequencing data will aid in understanding the efficacious actions of phytochemicals and their bioactive metabolites. These well-known characteristics of phytochemicals and phytochemical-metabolism can limit our in vitro interpretations. However, it is also not uncommon that in vitro findings correlate to mechanistic actions of select phytochemicals in vivo. For example, our lab and others found that emodin inhibited class I and II HDAC activity [10,15] and activated Sirt3 [38] in both cell culture and animal models of cardiac hypertrophy. While phytochemical distribution to tissues/cells of interest at bioactive/pharmacological concentrations may be a large hurdle, several technologies are being developed such as phytochemical-loaded nanoemulsions. For example, the aglycone of emodin does not under normal conditions distribute considerably to the heart; however, an emodin-loaded nanoemulsion significantly increased distribution of emodin to the heart of rats [63]. It will be interesting to see how increasing phytochemical bioavailability through different technologies affects the mechanistic actions of phytochemicals in vivo and how these actions differ from their unaltered parent compounds.

## 4. Materials and Methods

### 4.1. Chemicals and Reagents

The following dietary compounds were purchased from Selleckchem: apigenin (Selleckchem, Cat#- S2262), baicalein (Selleckchem, Cat#- S2269), baicalin (Selleckchem, Cat#- S2268), berberine hydrochloride (Selleckchem, Cat#- S2271), Cat#- S2268), caffeic acid (Selleckchem, Cat#- S2277), dihydromyricetin (Selleckchem, Cat#- S2399), emodin (Selleckchem, Cat#- S2295), (−)-epigallacatechin gallate (Selleckchem, Cat#- 2250), gossypol acetate (Selleckchem, Cat#- S2303), hematoxylin (Selleckchem, Cat#- S2384), indirubin (Selleckchem, Cat#- S2386), kaempferol (Selleckchem, Cat#- S2314), luteolin (Selleckchem, Cat#- S2320), morin hydrate (Selleckchem, Cat#- S2325), myricetin (Selleckchem, Cat#- S2326), myricitrin (Selleckchem, Cat#- S2327), palmatine chloride (Selleckchem, Cat#- S2397) and quercetin dihydrate (Selleckchem, Cat#- S2347).

### 4.2. Neonatal Rat Ventricular Myocyte (NRVM) Culture

Cardiac ventricles from one- to three-day old Sprague-Dawley rats (Charles River, Reno, NV, USA) were excised and minced before being digested in a calcium- and bicarbonate-free Hanks HEPES (CBFHH) solution containing trypsin (Gibco Life Technologies, Waltham, MA, USA) and DNaseII from bovine (Sigma-Aldrich, St. Louis, MO, USA). Isolated ventricular myocytes were then plated overnight in gelatin-coated (0.2%, Sigma-Aldrich) cell culture dishes (6-wells and 100 mm dishes) containing Minimum Eagles Medium (MEM, Genesee Scientific, San Diego, CA, USA) with 10% calf serum, 2 mM L-glutamine and penicillin-streptomycin. The next day, NRVMs were washed with Dulbecco’s Modified Eagles Medium (DMEM) before adding DMEM media containing Nutridoma-SP (Roche Applied Science, Penzberg, Germany). Finally, NRVMs were spiked with vehicle control (Veh; DMSO) or one of two hypertrophic agonists, phenylephrine (PE, 10 μM, Tocris Bioscience, Bristol, UK) or phorbol 12-myristate 13-acetate (PMA, 50 nM, Sigma-Aldrich), and co-spiked with one of the 18 dietary compounds listed above at 10 μM, except for apigenin which was dosed at 50 μM for 48 h prior to immunohistochemistry, cell viability, immunoblotting, RNA-seq and qPCR. Incubation time was chosen as 48 h is sufficient to see increased hypertrophy and molecular changes in response to PE or PMA [64]. Phytochemical dosing was chosen based on previously published work from our lab [10], showing the IC_50_ for these compounds on histone deacetylase activity, which is known to drive global changes in gene expression.

### 4.3. Immunohistochemistry

For immunohistochemistry experiments, a previously characterized protocol was followed which will be briefly described here [64]. After experimental treatment periods, NRVMs plated in 6-well dishes were washed with phosphate buffered saline (PBS, 7.6 pH) and then fixed in 4% paraformaldehyde at room temperature for 20 min. Next, a PBS solution containing bovine serum albumin (BSA, 3%, Fisher Bioreagents, BP1605) and Nonidet NP-40 (0.1%, Sigma-Aldrich IGEPAL CA-630) was used to permeabilize NRVM membranes. NRVMs were then incubated for two hours in a primary antibody cocktail containing antibodies for the hypertrophic marker, atrial natriuretic factor (ANF, 1:1000, Phoenix Pharmaceuticals, H-005-24) and the sarcomeric protein, α-actinin (1:750, Sigma-A7811). NRVMs were then incubated for one hour in a secondary antibody cocktail (goat anti-rabbit Cy3, Jackson ImmunoResearch; donkey anti-mouse FITC, Jackson ImmunoResearch, West Grove, PA, USA) before being washed in PBS and briefly exposed to Hoechst (10 μM, Invitrogen H3570). The EVOS FL Cell Imaging System (Thermo Fisher Scientific, Waltham, MA, USA) with DAPI, GFP and RFP imaging cubes were used to image nuclei, cardiomyocyte sarcomeres and the perinuclear expression of ANF, respectively, at a 20× objective. Twenty images were captured per well, resulting in 60 images per treatment, which were then used to analyze cell area via ImageJ software (NIH, Bethesda, MD, USA).

### 4.4. Cell Viability

NRVMs in gelatin-coated 96-well plates were spiked with either dimethyl sulfoxide (DMSO, veh, Pharmco-AAPER, St. Louis, MO, USA) or phenylephrine (PE, 10 μM, Tocris Bioscience,) and then co-treated with either Apigenin, Baicalein, Berberine Hydrochloride, Emodin, Luteolin or Quercetin dihydrate before incubated at 37 degree Celsius for 48-h per the experimental treatment period. Next, Invitrogen alamarBlue™ HS Cell Viability Reagent, a resazurin-based reagent that is reduced to the highly fluorescent resorufin in healthy cells, was used as described by the manufacturer instructions. Briefly, alamarBlue™ was added to each well at a ratio of 10:1 media:alamarBlue™ and NRVMs were incubated for one hour. BioTek Synergy was then used to measure fluorescence with a 530 nm excitation filter and a 590 nm emission filter. Results were normalized to DMSO-spiked NRVMs. 

### 4.5. Immunoblotting

After experimental treatments and incubation periods, NRVMs were washed with ice cold PBS and lysed with PBS containing 300 mM NaCl, 0.5% Triton-X and HALT™ protease/phosphatase inhibitors. NRVM lysates were sonicated and centrifuged to then separate the supernatant and pellet. The supernatant was isolated and used for immunoblotting experiments. BCA reagents were used to measure protein concentration of all samples as to normalize to 10 μg per sample for SDS-PAGE. Samples were resolved with SDS-PAGE and transferred onto a nitrocellulose membrane which was then incubated overnight in a primary antibody cocktail of BSA (2.5%) in 1x Tris-Buffered Saline and Tween^®^ (TBST). The next day, membranes were washed in TBST prior to being exposed to horseradish peroxidase-conjugated secondary antibodies (Southern Biotech, Birmingham, AL, USA). Finally, SuperSignal West Pico Chemiluminescence System (Thermo Fisher Scientific) was used on membranes prior to exposure on a ChemiDoc XRS+ Imager (BioRad, Hercules, CA, USA).

### 4.6. RNA Sequencing

NRVMs were lysed with QIAzol (Qiagen, Hilden, Germany) and RNA was isolated from treatment groups (*n* = 5) per the manufacturer’s instructions. Prior to synthesizing cDNA libraries, RNA integrity (RIN) and concentrations were analyzed by the Nevada Genomics Center using an RNA Agilent Bioanalyzer which confirmed RIN values were greater than 8.0. Following the Illumina’s protocol, the TruSeq Stranded Total RNA containing Ribo-Zero Human/Mouse/Rat kit (Illumina) was used to synthesize 1000 ng RNA into cDNA libraries. Libraries were then validated using the DNA 7500 chip and then sequenced using the NextSeq 500.

We used the Salmon tool (PMID: 28263959) to quantify the gene expression from the raw sequencing data, using the Ensembl rat gene annotation (Rnor_6.0). Transcript per million reads (TPM) was used to measure the gene expression level. The *edgeR* algorithm (PMID: 19910308) was applied to compare the groupwise gene expression pattern. We used the *TMM* algorithm implemented in the edgeR tool to perform reads count normalization and effective library size estimation. Groupwise differential expression was estimated by the likelihood ratio test implicated in the edgeR tool. The genes with false discovery rate (*FDR*) < 10% and fold change (*FC*) > 1.5 were deemed differentially expressed. Heatmaps were generated by the heatmap.2 function in the Rgplots package, with hierarchical clustering computed using Euclidean distance. For Heatmaps the gene expression level was scaled for each gene.

### 4.7. Real-Time qPCR

RNA was isolated as described above and cDNA was reverse transcribed from 500 ng RNA using the Verso cDNA Synthesis Kit (ThermoFisher Scientific). To determine mRNA expression, quantitative real-time polymerase chain reaction (qPCR) was performed using Apex qPCR Master Mix (Genesee Scientific, 42–120) and primers purchased from IDT (Table 2). 18S rRNA served as the endogenous control, particularly as we noted no change in mRNA expression >0.5 cycles (Ct) when using the 2^−ΔCt^ method in these analyses. We have previously published that endogenous controls with a Ct ≤ 0.5 are suitable for assessing relative gene changes [65]. To examine relative changes in our genes of interest, we used the 2^−ΔΔCt^ method.

### 4.8. Statistical Analysis

A minimum of three experiments with an *n* = 3 per experimental treatment group was performed and data quantified. One-way ANOVA with Tukey’s post-hoc was performed unless otherwise specified using GraphPad7 (GraphPad Software, La Jolla, CA, USA). *p* < 0.05 was considered significant.

## 5. Conclusions

In conclusion, apigenin, baicalein, BHCl, luteolin, emodin and QD all blocked PE- and PMA-induced cardiomyocyte hypertrophy. Transcriptomic and signal transduction analysis demonstrated that four of these dietary compounds impacted cardiac muscle cell enlargement through overlapping and divergent pathways. As expected, these data demonstrate that phytochemicals regulate a myriad of actions within the cell and are not limited to one mechanistic outcome (e.g., oxidation-reduction). However, these compounds also regulated similar pathways that are critical for cardiac enlargement and suggest that common targets of phytochemicals explain similarly observed phenotypes (i.e., anti-hypertrophic). Future studies examining these common targets in response to these compounds as well as in vivo analysis of these compounds and their metabolites are currently underway and will add to our knowledge for how these select phytochemicals and their metabolites regulate cardiac enlargement in pre-clinical models of heart failure. Lastly, much of our focus, to date, has been on examining individual phytonutrients in the prevention of heart enlargement. However, consumption of whole foods would be expected to contribute to cooperative, synergistic as well as antagonistic actions of phytochemicals as anti-hypertrophic agents. As such, it is important to note that four of our anti-hypertrophic compounds are found in common foods within our diet, while two are less common in our diet and thus more likely to be found in medicinal medicines or dietary supplements (Table 3). Therefore, work examining how phytonutrients interact in combination, or within the whole food, in vitro and in vivo is sorely needed and would increase our understanding for the protective actions of fruits and vegetables.

## Figures and Tables

**Table 1:** Dietary inhibitors of Cardiac hypertrophy.

Bioactive Food Compound	% Inhibition Cell Size	
	PE	PMA
Apigenin	100	91
Baicalein	100	100
Baicalin	N.I.	N.I.
Berberine Hydrochloride	100	52
Caffeic acid	N.I.	N.I.
Dihydromyricetin	N.I.	N.I.
Emodin	100	78
Epigallocatechin Gallate	48	14
Gossypol	Toxic	Toxic
Hematoxylin	N.I.	76
Indirubin	38	N.I.
Kaempferol	N.I.	30
Luteolin	62	40
Morin hydrate (Aurantica)	N.I.	26
Myricetin (Cannabiscetin)	Toxic	Toxic
Myricitrin (Myricitrine)	N.I.	40
Palmatine	81	N.I.
Quercetin dihydrate (Sophoretin)	45	42

**Table 2:** Primers for qPCR Validation.

	Forward Primer	Reverse Primer
ANP (Atrial Natriuretic Peptide)	GCCGGTAGAAGATGAGGTCAT’	GCTTCCTCAGTCTGCTCACTCA
BNP (Brain Natriuretic Peptide)	GGTGCTGCCCCAGATGATT	CTGGAGACTGGCTAGGACTTC
IL-6 (Interleukin-6)	CTTCACAAGTCGGAGGCTTAAT	GCATCATCGCTGTTCATACAATC
IL-1α (Interleukin-1α)	CAGATGGGCTAACTAAGGGATAAG	AGAAGGTGCACAGTGAGATAAG
IL-1β (Interleukin-1β)	CTTCCTAAAGATGGCTGCACTA	CTGACTTGGCAGAGGACAAA
CXCL2 (CXC Chemokine Ligand 2)	GGCGGGACGACTGTTATTT	CACTGTGCCTTACAGAGAAGAC
CXCL6 (CXC Chemokine Ligand 6)	GCTACGCTGTGTTTGCTTAAC	GCAGGGATCACCTCCAAATTA
CCL3 (CC Chemokine Ligand 3)	GAGATTAGAGGCAGCAAGGAA	CTTGGCAGCAAACAGCTTATAG
CCL6 (CC Chemokine Ligand 6)	GGGCTCATACAAGATACGGTAAA	CATGGGATCTGTGAGGCATAG
18S rRNA	GCCGCTAGAGGTGAAATTCTTA	CTTTCGCTCTGGTCCGTCTT

**Table 3:** Anti-hypertrophic compounds and their dietary source.

Phytochemical Class	Compound	Dietary Source
Flavonol	Quercetin	Teas, peppers, wines, onions, berries, apples
Flavone	Apigenin	Citrus, onions, celery, chamomile tea
	Luteolin	Celery, parsley, broccoli, onions, carrots, peppers, cabbages, apples
	Baicalein	*Scutellaria baicalensis*
Flavanolol	Dihydromyricetin	*Ampelopsis grossedentata* leaves and stems
Quinone	Emodin	Rhubarb, aloe vera, buckthorn, knotweed, fo-ti root, Cabbage, Beans
Alkaloid	Berberine Hydrochloride	*Hydrastis canadensis, Coptis chinensis, Berberis aquifolium, Berberis vulgaris, Berberis aristata*

## Data Availability

All supplemental tables can be found at MDPI. In addition, RNA-seq data will be made freely available to NCBI. Our data will also be made available to any investigator upon request.

## Supplementary Materials

The following are available online, Figure S1: Phytochemicals did not alter total protein expression in NRVMs, Table S1: Differentially regulated genes in response to pathological cardiac hypertrophy and phytochemicals treatment in neonatal cardiac myocytes.
