# Supplementary figures and images for: Divergent and Overlapping Roles for Selected Phytochemicals in the Regulation of Pathological Cardiac Hypertrophy
Source: Molecules. 2021 Feb 24;26(5):1210. doi: 10.3390/molecules26051210 (PMC7956446; doi:10.3390/molecules26051210)

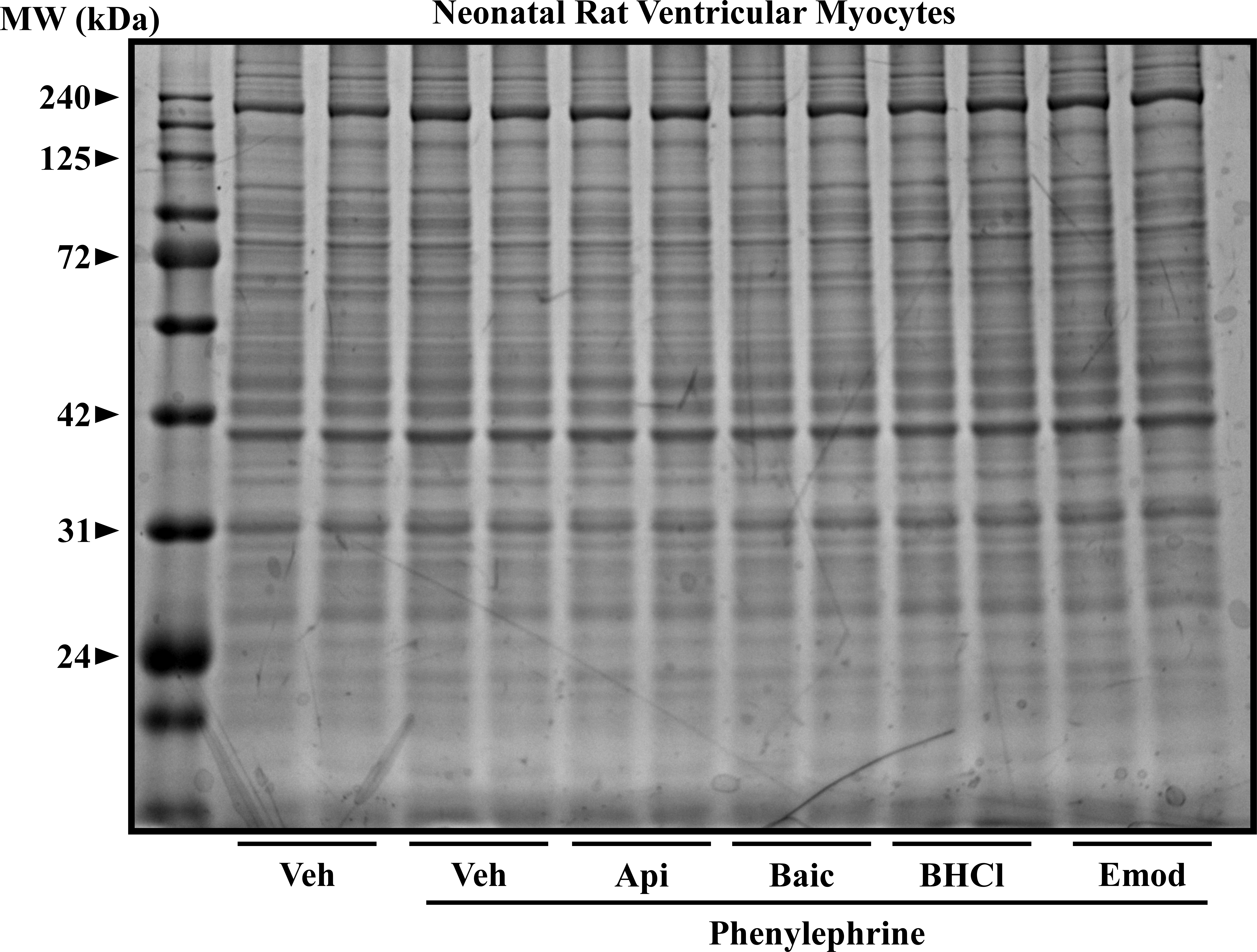

Supplement: Supplementary file 1 [file molecules-26-01210-s001.zip › SupplementalFigure_1.tif]
